# Efficacy and safety of Transcranial Direct Current Stimulation (tDCS) on cognitive function in chronic schizophrenia with Tardive Dyskinesia (TD): a randomized, double-blind, sham-controlled, clinical trial

**DOI:** 10.1186/s12888-023-05112-0

**Published:** 2023-08-24

**Authors:** Yue Zhou, Xingzhi Xia, Xueli Zhao, Ruchang Yang, Yuxuan Wu, Junjun Liu, Xiaoli Lyu, Zhe Li, Guangya Zhang, Xiangdong Du

**Affiliations:** 1 Xuzhou Medical University, Xuzhou, China; 2 Suzhou Guangji Hospital, The Affiliated Guangji Hospital of Soochow University, Suzhou, China; 3 Medical College of Soochow University, Suzhou, China; 4 Nanjing Meishan Hospital, Nanjing, China; 5 Affiliated WuTaiShan Hospital of Medical College of Yangzhou University, Yangzhou, China

**Keywords:** tDCS, Randomized Controlled Trial, Chronic Schizophrenia, Tardive Dyskinesia, Pattern Recognition Memory, Intra/Extradimensional Set Shift

## Abstract

**Objective:**

Previous studies have shown that transcranial direct current stimulation(tDCS) led to an improvement of cognitive function in patients with schizophrenia, but rare study has explored the effect of tDCS on long-term hospitalized chronic schizophrenia with tardive dyskinesia (TD). The present research explored if cognitive function in patients with long-term hospitalized chronic schizophrenia with TD could be improved through tDCS.

**Methods:**

This study is a randomized, double-blind, sham-controlled clinical trial. Of the 52 patients, 14 dropped out, and 38 completed the experiment. Thirty-eight patients on stable treatment regimens were randomly assigned to receive active tDCS(n = 21) or sham stimulation(n = 17) on weekdays of the first, third, and fifth weeks of treatment. Patients performed the Pattern Recognition Memory (PRM) and the Intra/Extradimensional Set Shift (IED) from the Cambridge Neuropsychological Test Automated Battery (CANTAB) at baseline and the end of week 3, week 5. Clinical symptoms were also measured at the baseline and the fifth week using the Scale for the Assessment of Negative Symptoms (SANS) and the Positive and Negative Syndrome Scale (PANSS). Side effects of tDCS were assessed with an experimenter-administered open-ended questionnaire during the whole experiment.

**Results:**

There were no significant differences in PRM and IED performance metrics, SANS total score and PANSS total score between active and sham tDCS groups at the end of week 5 (*p* > 0.05). Furthermore, there was a significant difference in the adverse effects of the tingling sensation between the two groups (*p* < 0.05), but there was no significant difference in other side effects (*p* > 0.05).

**Conclusion:**

According to these findings, no evidence supports using anodal stimulation over the left dorsolateral prefrontal cortex to improve cognitive function in patients with long-term hospitalized chronic schizophrenia with TD.

## Introduction

Schizophrenia is a severe chronic psychiatric disorder that originates from disruption of brain development caused by genetic or environmental factors [1]. The median incidence of schizophrenia was 15.2/100,000 people, with a lifetime prevalence of about 1% [2, 3]. It is characterized by a series of psychotic symptoms, such as hallucinations, delusions, and cognitive impairment [4]. Cognitive impairment is a stable feature of schizophrenia associated with patients’ functional status [5]. It is supposed to be the result of neurodegenerative changes, and neurodevelopmental abnormalities have an impact on cognitive impairment in schizophrenia [6]. A recent study showed that cognitive impairment in patients with schizophrenia manifested in verbal and working memory, processing speed, verbal fluency, reading ability, and non-verbal reasoning [7]. In a meta-analysis study [8], compared to bipolar patients, patients with schizophrenia have more severe cognitive dysfunction, particularly in attention and social cognition. Since the serendipitous discovery of chlorpromazine more than 50 years ago, almost all antipsychotic drugs available in the clinical setting for schizophrenia work via dopamine D(2) receptor blockade [1]. However, it may lead to clinical responses, extrapyramidal side effects, and hyperprolactinemia [9].

Tardive dyskinesia(TD) is caused by the long-term use of dopamine blockers [10]. It is a severe motor adverse event due to antipsychotic medication, characterized by involuntary athetoid movements of the trunk, limbs, and/or orofacial areas [11]. The prevalence of TD is reported to be 25.3%, with a 30.0% incidence of TD due to first-generation antipsychotics (FGAs) and 20.7% of TD due to second-generation antipsychotics (SGAs) [12]. The incidence of TD has decreased since SGAs became available [13]. Despite this, a previous study reported that the reduction of TD with SGAs appears unlikely to meet standards for cost-effectiveness [14]. A large proportion of patients with chronic schizophrenia suffer from various degrees of TD. Even the newer antipsychotics constitute a risk for TD [15]. As well, the more severe TD is, the faster the decline in the quality of life, placing a heavy financial burden on patients and their families [14, 16]. Studies have shown that patients with moderate or severe TD had 12.3% lower Quality of Life Interview scores than those without this side effect [14]. Moreover, research has proven that TD was implicated in greater cognitive impairment in patients with schizophrenia compared to those without TD [17]. Although some earlier studies suggested the withdrawal of antipsychotics as the treatment for TD, no confident statement can be made about the effectiveness of the withdrawal of anticholinergics in benefiting patients with TD [18–20]. So some researchers believe that preventing TD is of primary importance [21]. In 2017, FDA approved vesicular monoamine transporter type 2(VMAT2) inhibitors, deutetrabenazine, and valbenazine for the treatment of patients with TD [22]. A growing body of research is beginning to focus on the treatment of cognitive impairment in patients with schizophrenia with TD.

Noninvasive brain stimulation (NIBS) has been proposed as a new therapeutic option for treating cognitive deficits in schizophrenia [23]. Transcranial direct current stimulation(tDCS), a novel noninvasive brain stimulation technique, consists of two relatively large electrodes: the cathode and the anode. In most current studies, researchers choose the left dorsolateral prefrontal cortex (DLPFC) as the anode [24–27]. It has been found to improve cognitive function and symptoms of neuropsychiatric disorders such as Parkinson’s disease (PD), essential tremor (ET), dystonia, and progressive supranuclear palsy (PSP) [28]. It can also regulate cognitive functions such as working memory and learning processes in different ways [29, 30]. A study has also found it improves cognitive function in Alzheimer’s patients [31], however, one meta-analysis noted that its efficacy is controversial and needs further study [32]. Previous studies have shown significant effects of different cerebellar neurostimulation techniques on movement disorders [28]. Most tDCS treatments choose the left dorsolateral prefrontal cortex (DLPFC) [33, 34] as the anodal stimulation site to enhance the excitability of DLPFC to modulate the cerebral circuitry and choose the contralateral upper orbital border area as the cathodal. A case study of rTMS for TD, which also used the left DLPFC as the anodal stimulation site, found short-term improvement in TD symptoms [35]. A pilot study [36] in 2022 noted tDCS as a potential treatment approach for TD in schizophrenia disease. However, some investigators have suggested that their efficacy is variable and that the heterogeneity of efficacy results is related to the individual, clinical conditions, and different devices and parameters [37–39]. Given the results of the current study, no clinical indications have reached level a (definitive efficacy) recommendations according to the guidelines published by the European expert association [40]. In recent years there has been an increasing number of applications regarding tDCS in psychiatry. Nevertheless, most of the patients in this study are primarily single disorders, such as schizophrenia and Alzheimer’s disease, while the novelty of our study is that the included patients were schizophrenic patients with TD, intending to study the effect of tDCS for improving cognitive function and psychiatric symptoms in schizophrenic patients with TD.

## Materials and methods

### Subject

This study is a double-blinded, parallel-group, randomized controlled trial that compares an intervention group receiving tDCS with a parallel control group not receiving it. A-priori sample size is calculated with G*Power. We conservatively assume a small to medium effect size of f = 0.3, with an α-level of 0.05 and a power-level of 0.80. We have two groups and four measurements, thus 18 patients per condition. We recruited 52 patients with chronic schizophrenia who had been hospitalized for a long time at the Jiangsu Province Suzhou Guangji Hospital, Suzhou Social Welfare Institute, Taicang Third People’s Hospital, Nanchong Psychosomatic Hospital, Wuxi Mental Health Center in mainland China from July 2017 to December 2019.

The inclusion criteria included: (1) Meets the diagnostic criteria for schizophrenia according to the Diagnostic and Statistical Manual of Mental Disorders, 4th Edition (DSM-IV)^;^ (2) aged between 18 years and 70 years;(3) cumulative use of antipsychotics for at least one year;(4)TD status was determined diagnosis of TD based on the Schooler-Kane criteria [41], that is, patients who had at least one Abnormal Involuntary Movements Scale (AIMS) item rated > 3(moderate) or at least two AIMS items rated ≥ 2 (mild) were considered as having a diagnosis of TD;(5)right-handed;(6)patients volunteered to participate in this study. The exclusion criteria included: (1)movement disorders such as schizophrenic stereotypes, Parkinson’s disease(PD), Tourette’s syndrome(TS), Huntingtong’s chorea, tooth loss, and other oral diseases caused by abnormal movements of the mouth and face;(2)with color blindness/weakness, stuttering, deafness.

The study was approved by the Institute Review Board Committees of Suzhou Guangji Hospital. The trial was registered with ClinicalTrials.gov on 13/04/2018. (No.NCT03497013).

### Design

The patients received 15 consecutive sessions of either active or sham tDCS on all weekdays of the first, third, and fifth weeks (15 sessions in total). All patients received antipsychotic medications, and their medications remained unchanged during treatment. Clinical assessments were performed at baseline and at the end of week 5. Cognitive tests were performed at baseline, at the end of week 3, and week 5.

### Randomization and blinding

Consistently trained physicians assess enrolled patients. A tDCS operator used SPSS, with a default fixed value of 2,000,000, to generate a table of random numbers. Then delivered either tDCS or sham according to a randomization list in a 1:1 ratio. Only the tDCS operators knew the grouping of each patient and revealed it to researchers after all patients had completed the last assessment. Hence, both patients and assessors are blinded to the treating conditions.

### Active and sham tDCS

tDCS is a Transcranial Electrical Stimulation (tES) technique that was delivered by Soterix Medical 1 × 1 Low-Intensity Transcranial Electrical Stimulator (tES) Model 2001. The stimulation site is the left dorsolateral prefrontal cortex (DLPFC) and contralateral upper orbital border area. According to previous research, we choose the left dorsolateral prefrontal cortex (DLPFC) as the anodal point and the contralateral upper orbital border area as the cathodal point. The current is two mA continuous, direct current; the plastic electrode piece is placed in a sponge (5 cm *7 cm) soaked in normal saline, the electrode should not be exposed during placement. Stimulation is done for 30 min. For sham stimulation, the treatment parameter setting and evaluation tools were the same as the active group, except the current will stop after 30 s from stimulation, so the same sensation as in active group (tingling of the skin) was induced.

### Psychopathological measures

General psychopathology was assessed using the Positive and Negative Syndrome Scale (PANSS). Negative symptoms were also assessed with the Scale of Assessment of Negative Symptoms (SANS), which consists of 19 items assessing five symptoms of the negative dimension: affect flattening, alogia, avolition-apathy, anhedonia-asociality, and poor attention. Two clinical psychiatrists blinded to treatment condition (real vs. sham tDCS) assessed PANSS and SANS scores at baseline and the end of week 5. Inter-rater reliability was satisfactory for both tests (κa = 0.88 for PANSS and κa = 0.86 for SANS).

### Cognitive performance

Cambridge Neuropsychological Test Automated Battery (CANTAB)is a computerized, language and culture-free neuropsychological cognitive testing tool that is non-verbal in the vast majority of tasks, including geometric and simple patterns and machine sounds. CANTAB is considered by researchers to be the latest generation of neuropsychological cognitive detection tools and has been widely used in the field of neuropsychiatric research abroad [42]. This study used the Pattern Recognition Memory (PRM) and the Intra/Extradimensional Set Shift (IED) modules in the CANTAB to test their visual short-term recognition memory and executive function.

PRM: The test is divided into two stages. The first stage is the immediate mode, the center of the screen at a rate of 1 per second after the presentation of 12 graphics. In the re-recognition stage, the patient is required to choose between the pattern that has been seen and a new shape, select the pattern seen; the second stage is the delay mode, the center of the screen will display 12 new graphics, the patient also needs to carefully observe and remember, after 20 min, and then let the patient select the pattern seen. PRM primarily tests visual short-term recognition memory. The test indicators are immediate mode accuracy and delay mode accuracy. The higher the correct rate, the better the patient’s visual recognition memory.

IED: The quiz is a test of the subject’s ability to acquire and convert rules and the maintenance, conversion, and flexibility of attention. During the operation, two graphics will appear in the middle of the computer screen, one is right, and the other is wrong. The emergence of the graph is a certain law. The rules or graphs will change after six consecutive correct clicks on the graph. The first time the subject needs to produce the correct graph, and then the subject needs to summarize and find out what the law is, the correct graph is selected. The transformation of the rule is from the initial internal dimension to the external dimension. Subjects must meet the prescribed criteria at each stage to move on to the next stage. The test indicators are the number of errors in the previous period of external conversion, the number of errors in the external conversion period, the number of completed stages, and the total number of errors. The number of errors in the external dimension conversion stage reflects the patient’s ability to pay attention to the conversion of the complete set, which is used to illustrate the flexibility and execution control problems when the task needs to be changed, and the total number of corrected errors includes the number of errors in the external dimension conversion stage and the number of completed stages, which is a comprehensive indicator of the quality of the attention set conversion task.

### Stopping guidelines

The study will be stopped once the following conditions occur: 1. Serious adverse events and side-effects occur that make continuing the study difficult;2. Patients or their families do not wish to continue to participate in the trial and withdraw their informed consent.

### Statistics

All the analyses were completed in SPSS version 23.0. Continuous baseline variables were compared between active and sham tDCS groups by independent sample t-test. Categorical variables were presented as frequency and compared by χ2 test. The primary objective of this study was to evaluate the effect of tDCS on cognitive function in chronic schizophrenia with TD. So the principal outcome was analyzed by repeated-measures analyses of variance with measurement time (baseline and weeks 3 and 5) as the within-group factor and active versus sham tDCS as the between-group factor. If the time × group interaction was significant, analysis of covariance (ANCOVA) was used to test for differences between groups at the end of weeks 3 and 5, with baseline score as the covariate. If the interaction was not significant, no further statistical tests were performed. The independent samples t-test was used to analyze changes in PANSS and SANS total scores. *p* ≤ 0.05 (two-tailed) was considered significant for all tests.

## Results

### Socio-demographical and clinical characteristics of the patients

As shown in Tables 1 and 38 patients were enrolled in the research, randomized into active groups (21 cases) and sham groups (17 cases) at baseline. There were no statistically significant differences in the general demographic characteristics of the two groups, such as sex, age, course, and educational attainment (*p* > 0.05). There was no statistically significant difference in the equivalent dose of chlorpromazine between the two groups (*p* > 0.05).

**Table 1:** Socio-demographical and clinical characteristics of the patients

	active group (n = 21)	sham group (n = 17)	χ2 */t*	*p*
gender			0.003	0.955
male (%)	15(39.47)	12(31.58)		
female (%)	6(15.79)	5(13.16)		
age, y	56.71±9.31	54.65±9.43	0.676	0.503
duration of illness, y	27.81±10.98	22.18±10.74	1.588	0.121
educational background			6.512	0.075
bachelor’s degree or above	1(2.63)	0		
senior high school	2(5.26)	7(18.42)		
junior high school	11(28.95)	4(10.53)		
primary school	7(18.42)	6(15.79)		
chlorpromazine equivalent dose	400.00(245.00,600.00)	400.00(250.00,500.00)	-0.399	0.690

### Analysis of PRM and IED indicators in active group and sham group

The ANOVA results showed that the main effect of group was not significant, the main effect of time was not significant, and the interaction effect between time and group was not significant (*p* > 0.05). The simple effect analysis results showed that the active group compared to sham had no significant PRM and IED scores at the third week of treatment and at the end of treatment (*p* > 0.05). (Table 2)

**Table 2:** Analysis of PRM and IED indicators in active group and sham group

	baseline (n = 38)	week 3 (n = 38)	week 5 (n = 38)	group F (*p*)	time F (*p*)	group x time F(*p*)
accuracy(immediate)/%				0.65 (0.43)	2.97 (0.06)	0.11 (0.88)
active group (n = 21)	55.9 ± 21.1	61.9 ± 17.2	64.7 ± 21.2			
sham group (n = 17)	51.5 ± 20.5	59.8 ± 18.2	59.3 ± 21.4			
accuracy(delay)/%				0.95 (0.34)	5.6 (0.10)	0.08 (0.91)
active group (n = 21)	46.0 ± 15.0	54.8 ± 14.8	50.0 ± 17.3			
sham group (n = 17)	51.0 ± 16.9	57.8 ± 11.2	53.9 ± 13.5			
IED pre-ED errors				1.34 (0.25)	1.85 (0.17)	0.99 (0.37)
active group (n = 21)	24.9 ± 18.3	22.4 ± 16.1	21.2 ± 16.1			
sham group (n = 17)	29.7 ± 18.2	32.8 ± 25.9	25.8 ± 21.4			
IED EDS errors				0.14 (0.71)	0.21 (0.78)	0.10 (0.90)
active group (n = 21)	13.3 ± 13.4	12.3 ± 12.6	12.5 ± 13.0			
sham group (n = 17)	11.6 ± 13.1	10.4 ± 13.0	12.3 ± 13.2			
IED stages completed				0.06 (0.80)	1.23 (0.30)	0.47 (0.61)
active group (n = 21)	4.7 ± 3.2	5.4 ± 2.7	5.0 ± 3.2			
sham group (n = 17)	4.8 ± 3.0	5.3 ± 2.9	5.7 ± 3.0			
IED total errors				0.01 (0.94)	1.46 (0.24)	0.44 (0.63)
active group (n = 21)	122.1 ± 77.0	107.2 ± 71.4	111.7 ± 76.7			
sham group (n = 17)	125.5 ± 67.7	116.8 ± 64.6	103.1 ± 71.9			

### Analysis of PANSS and SANSS scores in active and sham groups

Table 3 shows no statistically significant difference in the scores of the PANSS and SANS assessments in both groups(*p* > 0.05). There was also no statistically significant difference between the two groups in the PANSS and SANSS scale scores after the end of treatment at week 5(*p* > 0.05).

**Table 3:** Analysis of PANSS and SANSS scores in active and sham groups

	active group (n = 21)	sham group (n = 17)	t	*p*
baseline				
PANSS total score	62.05±10.552	61.00±9.823	0.314	0.756
SANS total score	53.57±16.663	50.18±16.753	0.623	0.537
week 5				
PANSS total score	61.24±13.616	56.00±10.724	1.293	0.204
SANS total score	52.05±18.513	49.12±13.874	0.541	0.592

### Adverse reactions to transcranial direct current stimulation

Compared with the sham group in Table 4, the difference in adverse reactions of a tingling sensation was statistically significant (*p* < 0.05), and the difference in other adverse reactions was not significant (*p* > 0.05).

**Table 4:** Adverse reactions to transcranial direct current stimulation

	active group (n = 21)	sham group (n = 17)	χ2	*p*
tingling	18	8	6.497	0.011*
itching	5	3	0.215	0.643
burning sensation	4	2	0.375	0.540
pain	2	2	0.050	0.823
fatigue	2	1	0.171	0.679

*p < 0.05

## Discussion

The important result of this study is that tDCS could not improve cognitive function in patients with long-term hospitalized chronic schizophrenia with TD. Conversely, in an earlier randomized controlled study [43], the total cognitive function scores, working memory, and attentional alertness scores of patients treated with tDCS were significantly higher than those in the pseudo-stimulus group. Similarly, previous studies [44, 45] have shown that tDCS can improve the patient’s working memory when using the left dorsolateral prefrontal cortex as an anode stimulation point. These results are inconsistent with this research. Some studies provided potential explanations for this discrepancy. First, the long duration of our participants may account for the difference. All our patients have been ill for over 20 years. The meta-analysis of older adults with schizophrenia demonstrated that long duration of illness has a strong relationship with worse cognitive [46]. In a prospective follow-up of cognitive dysfunction in patients with chronic schizophrenia [47], cognitive function significantly deteriorated over ten years. More impressively, a study reported that schizophrenia patients could develop additional cognitive impairment, including dementia [48]. It suggests that the degree of cognitive dysfunction in patients with schizophrenia correlates significantly with the disease’s duration. Second, the age of patients was taken into account in our study. The average age of the patients enrolled in our study was around 55. The meta-analysis also mentioned that age is related to cognitive [46]. Compared with younger, older patients with schizophrenia have more significant deficits in nearly all cognitive domains [49]. Third, patients enrolled in the study are accompanied by TD. Based on a prospective follow-up study [47], patients with TD have poorer cognitive function than those without. In a review of cognitive rehabilitation in patients with schizophrenia [50], experts point out that future studies need to investigate the mechanisms and various mediators underlying the relationship between cognitive function and functional outcomes. With more comprehensive cognitive and social cognitive programs, we can improve the cognitive and functional outcomes of patients with schizophrenia. Therefore, some experts suggested that we should regularly assess the effects of TD on psychiatric disorders [51] as TD may to some extent reflect the severity of cognitive dysfunction.

In this study, we used PANSS and SANS to assess the psychiatric symptoms of patients. Based on the result, we found that the psychiatric symptom scores of the two groups all improved after tDCS. However, there was no significant difference in psychiatric symptom scores after treatment between the two groups of patients. However, in a 2020 randomized clinical trial [24] of 100 patients with schizophrenia, the researchers divided 100 people into a tDCS group and a sham group. Patients receiving active tDCS showed a significantly greater improvement in PANSS score compared with those receiving the sham procedure. In a randomized controlled, double-blind experiment [52], the researcher showed the efficacy of bi-anodal transcranial direct current stimulation (tDCS) over the prefrontal cortex (PFC) regions with extracephalic reference placement in improving negative symptoms in schizophrenia. Post-hoc analyses showed tDCS rapidly reduced PANSS total score with sham treatment. Several reasons may account for this difference. One is that our patients are long-term hospitalized chronic patients with schizophrenia. Another reason could be that tDCS use different parameters. In a systematic review and meta-analysis of the effects of tDCS, suggests that for anodal tDCS, the duration of the stimulation and the task used to probe memory moderated the effectiveness of tDCS. For cathodal tDCS, the site of stimulation was a significant moderator [53]. Thus in the double-blind experiment [52], their tDCS used a double anode, which is not the same as our experimental setup. This also makes us pay attention to the tDCS usage mechanism and the setting of its parameters. Further, the efficacy of different tDCS regimens for the psychiatric symptoms of schizophrenia requires further investigation in larger clinically heterogeneous populations. Finally, due to our small sample size, the sample size should be expanded in future studies to validate the results of this study.

At present, the mechanism of tDCS in the treatment of cognitive function in patients with schizophrenia is not entirely clear. Current research suggests that mechanisms posited to underlie its effects include modification of cortical excitability and neural plasticity [54]. Available data from studies in animals and humans suggests that the current strengths typically administered to humans modulate neural activity by way of changes in electrical fields and neural oscillations [55]. Electric fields induced by tDCS can augment neurite outgrowth and axonal regeneration [56]. The direction of the axonal processes is important for the effect of electrical stimulation because the direction of the current affects the neural effect [54]. Synaptic plasticity is thought to be central to brain plasticity, making synapses a natural focus for long-lasting tDCS effects. Furthermore, in human and animal studies, changes in synapse-mediated evoked responses are considered reliable markers of long-term plasticity changes that can support long-lasting behavioral or clinical changes [57]. In addition, tDCS may induce nonsynaptic effects that produce its lasting sequelae, as it affects the entire axon [58]. Earlier studies [59, 60] argue that the pathophysiology of negative symptoms has been associated with decreased activity of the prefrontal cortex (PFC). Equally, recent findings [61] suggest that frontotemporal tDCS with the cathode placed over the left temporoparietal junction (TPJ) and the anode over the left prefrontal cortex can alleviate treatment-resistant auditory verbal hallucinations in patients with schizophrenia. tDCS is divided into two types: anode stimulation and cathodic stimulation, which regulate the membrane potential by applying a weak current (0.5-2mA) to the scalp, which in turn affects cerebral cortex activity and induces changes in brain function [62]. Generally, cathodal stimulation decreases neuronal excitability in the targeted area, whereas anodal stimulation increases it [63]. However, various studies now suggest that the cerebellum may influence cognitive processes. For example, in the field of anatomy, many studies suggest that interconnections between prefrontal cortex regions and the cerebellum (i.e., brain-cerebellar pathways) may support cognitive [64]. In most clinical trials, only a limited range of intensities (1–2 mA) exists. A double-blind experiment with low-intensity (1 mA) tDCS [62]shows a failure of placebo control during 1 mA tDCS. Some scholars have pointed out the physics of tDCS dictates that current flow intensity in the brain (electric field) will increase linearly with applied current [65]. In recent years, there has been growing attention on dose control of tDCS. Several studies demonstrate [66–68] that simple dose responses do not affect the effectiveness of the dose given to tDCS. Understanding the dose response of tDCS in human applications is necessary for protocol optimization, including individualized doses that reduce outcome variability.

Previous studies [69–71] have shown that tDCS adverse effects are mostly mild, common in itching, burning, or headache, with no long-term effects, so tDCS is considered safe. This in accordance with our study (shown in Table 4). None of the patients in this study experienced severe adverse effects, and some patients only reported brief and mild tingling, itching, burning, pain, and other discomforts at the beginning of treatment.

## Limitation of the study

This study had several limitations. First, the study had a smaller sample size and was a multicenter-controlled study. Although there is no change in the evaluation of psychiatric symptoms and cognitive function in patients between different hospitals in this study, there is heterogeneity in patients in different regions and different hospitals, so there are differences in the baseline course of the two groups of patients. It will be supposed to expand the sample size in follow-up studies to explore further. Secondly, the CANTAB cognitive assessment model selected in this study is incomplete. There are only two dimensions of PRM and IED, and the assessment module should be added to explore the cognitive function effect of tDCS in patients with chronic schizophrenia from different cognitive dimensions, to provide new methods for the treatment of patients.

## Conclusion

The results failed in demonstrating that the tDCS was effective on visual short-term recognition memory, attention to the flexibility of the conversion, and executive function and psychiatric symptoms (PANSS and SANS total scores) in patients with chronic schizophrenia with TD, and patients treated with tDCS had a significant adverse effect of tingling.

## Data Availability

The data that support the findings of this study are available on request from the corresponding author upon reasonable request.
